# Positive association between heart dosimetry parameters and a novel cardiac biomarker, solubleST‐2, in thoracic cancer chest radiation

**DOI:** 10.1002/jcla.23150

**Published:** 2020-01-10

**Authors:** Zhi‐min Zeng, Peng Xu, Shan Zhou, Hai‐yang Du, Xiao‐liu Jiang, Jing Cai, Long Huang, An‐wen Liu

**Affiliations:** ^1^ Department of Oncology The second affiliated hospital of Nanchang University Nanchang China; ^2^ Jiangxi key laboratory of clinical translational cancer research The second affiliated hospital of Nanchang University Nanchang China

**Keywords:** cardiac biomarker, heart dosimetry parameters, radiation‐induced heart disease, radiotherapy, sST‐2

## Abstract

**Background:**

Early screening and diagnosis of radiation‐induced heart disease (RIHD) is difficult in patients with chest radiation exposure. sST‐2 is involved in myocardial stress or injury. We evaluated the relationship between heart dose parameters and sST‐2 changes in chest malignant tumor patients who received chest radiation.

**Methods:**

We prospectively collected thoracic malignancy cancer patients who had received chest radiotherapy. Heart dosimetry parameters were extracted from the treatment planning system. sST‐2 was measured at baseline, the middle stage, and after radiotherapy (recorded as pre‐ST‐2, mid‐ST‐2, and post‐ST‐2). sST‐2 change rate was calculated. Scatter plots showed the relationship between cardiac dose parameters and ST‐2 change rate. Multiple regression was used to analyze the relationship between cardiac dose parameters and ST‐2 change rate.

**Results:**

Totally, 60 patients were enrolled. The mean V_5_, V_10_, V_20_, V_30_, V_40_, and MHD was 60.93 ± 27.79%, 51.43 ± 25.44%, 39.17 ± 21.75%, 28.07 ± 17.15%,18.66 ± 12.18%, and 18.60 ± 8.63 Gy, respectively. The median M‐LAD was 11.31 (IQR 3.33‐18.76) Gy. The mean pre‐ST‐2, mid‐ST‐2, and post‐ST‐2 was 5.1 ± 3.8, 6.4 ± 3.9, and 7.6 ± 4.4, respectively. sST‐2 was elevated with thoracic irradiation (*P* < .001). Multivariate linear regression analyses showed that V_5_, V_10_, V_20_, and MHD were independently and positively associated with ST‐2 change rate (*β* = .04, .04, .04, and .10, respectively, all *P* < .05).

**Conclusion:**

Serum sST‐2 levels were elevated over time during radiotherapy. V_5_, V_10_, V_20_ and MHD were independently and positively associated with the elevated ST‐2 change rate.

**Abbreviations**
BNP: B‐type natriuretic peptide
DVH: dose‐volume histogram
IMRT: intensity‐modulated radiotherapy
IQR: interquartile range
LVEF: the left ventricle ejection fraction
MHD: mean heart dose
M‐LAD: mean dose of left anterior descending artery
RIHD: radiation‐induced heart disease
RT: radiotherapy
sST‐2: solubleST‐2
VMRT: volumetric‐modulated radiotherapy

## 1 INTRODUCTION

Thoracic radiotherapy (RT) is one of the main treatments for lung cancer,^1^, ^2^ esophageal cancer,^3^ and thymoma.^4^Cardiotoxicity is a serious problem threatening the survival and quality of life of patients undergoing thoracic radiotherapy.^5^ A large number of studies have shown that RIHD can occur in patients with breast cancer, childhood lymphoma, and other cancers decades after radiotherapy.^6^, ^7^, ^8^Until now, there was no standard diagnostic method for RIHD. Myocardial markers (including brain natriuretic peptide (BNP), pro‐BNP, and cTnI), echocardiography, and cardiac magnetic resonance are common methods for diagnosing RIHD.^8^, ^9^ Early screening and diagnosis of RIHD are difficult in patients with radiation exposure.

ST‐2 is a member of the interleukin‐1 receptor family and includes transmembrane (ST‐2L) and soluble ST‐2 isoforms (sST‐2).^10^ IL‐33 is a specific ligand of ST‐2L, forming the IL‐33/ST‐2 signaling pathway, which is involved in myocardial stress or injury.^11^, ^12^ Multiple studies revealed that elevated sST‐2 concentration is involved in various heart diseases, such as heart failure,^13^, ^14^, ^15^ atrial fibrillation,^16^ heart transplant recipients,^17^, ^18^ chronic kidney disease‐induced cardiac remodeling,^19^ and myocardial infarction.^11^, ^20^ The guidelines for heart failure management issued by ACCA/AHA in 2013 recommend soluble ST‐2 as an additional indicator of risk stratification in patients with acute and chronic heart failure.^13^

Heart radiation exposure can lead to vascular endothelial cell damage and vascular inflammatory reactions, resulting in interstitial ischemic fibrosis caused by thrombosis or inflammatory reactions. A study demonstrated that sST‐2 levels in workers from the nuclear industry were significantly higher (fivefold) than the control group without exposure history.^21^ When patients receive chest radiotherapy, the heart is exposed to large doses of X‐rays in a short duration, especially in central lung tumors and esophageal cancer. However, the effect of chest radiation on sST‐2 and whether changes in sST‐2 levels are associated with cardiac doses was unclear.

In this study, we examined sST‐2 levels in the serum of patients receiving high‐dose radiotherapy for thoracic malignancies. We evaluated early changes in serum sST‐2 levels during thoracic radiotherapy and determined associations between heart dosimetry parameters and ST‐2 change rate.

## 2 METHODS AND MATERIALS

### 2.1 Patients and study design

Patients with malignant thoracic tumors who underwent thoracic RT in the Department of Thoracic Oncology, Second Affiliated Hospital of Nanchang University from October 2016 to August 2018 were enrolled in this study. All patients had received thoracic irradiation, including radical radiotherapy, adjuvant radiotherapy, or palliative radiotherapy. Each patient underwent sST‐2 and BNP determination before RT (recorded as pre‐ST‐2 or pre‐BNP), in the middle of RT (recorded as mid‐ST‐2 or mid‐BNP), and after the end of RT (recorded as post‐ST‐2 or post‐BNP). Echocardiography was performed before and after RT (recorded as pre‐left ventricular ejection fraction (pre‐LVEF) or post‐LVEF).

Inclusion criteria were as follows: age >18 years, Eastern Cooperative Oncology Group performance status 0‐2, and adequate hematologic, hepatic, and renal function. Exclusion criteria were as follows: the presence of another primary cancer (excluding skin cancer beyond 5 years), thoracic radiation historically, malignant pericardial effusion, uncontrolled angina pectoris, myocardial infarction <3 months before enrollment, interstitial pneumonia, active lung fibrosis, or severe cachexia. All enrolled patients provided consent and the study was approved by the Ethics Committee of the Second Affiliated Hospital of Nanchang University.

### 2.2 Serum sST‐2 and BNP array

Blood samples were collected in tubes with EDTA and serum was separated by centrifugation for 10 min at 600 × *g*. The serum samples were stored at −80°C for later use. sST‐2was determined using a high‐sensitivity enzyme‐linked assay (ELISA) kit (Presage ST‐2 assay; Critical Diagnostics) according to the manufacturer's procedures. sST‐2 levels were evaluated after determining the optical density of the samples at 450 nm (Thermo Scientific Microplate Reader, Varioskan LUX). BNP was detected in our clinical laboratory collected in medical record system.

### 2.3 Cardiac echocardiography

Cardiac echocardiographic examinations were performed using GE Vivid (GE Healthcare, Vivid E9) by experienced physicians who were blinded to all treatment data. LVEF was collected before and after radiotherapy.

### 2.4 Irradiation

All patients received intensity‐modulated or volumetric‐modulated RT (IMRT or VMRT) using an Elekta linear accelerator (Elekta Versa HD) in a supine position fixed with mask or vacuum bag. The targets and organs at risk (OAR), including the heart, were contoured by the same physician, and the treatment plan was designed by a specific physician. The dose of normal tissues was constrained (lung of V_20 _<32%, V_30 _<20%, heart of V_40 _<30%). The dosage of RT was as follows: 5040‐6400 cGy/28‐32F for thymoma, 4500‐6720cGy/28‐32F for esophageal cancer, 4500 cGy/15F × BID or 5040‐6400 cGy/28‐32F for small cell lung cancer, and 5040‐6600 cGy/28‐32F for non‐small cell lung cancer. Heart dosimetry parameters, including V_5_, V_10_, V_20_, V_30_, V_40,_ the mean dose of the heart (MDH), and the mean dose of the left anterior descending coronary artery (M‐LAD), were extracted from the dose‐volume histogram (DVH) curves in the Monaco treatment planning system (Elekta Versa HD).

### 2.5 Statistical analysis

Data are presented as mean ± SD or median (interquartile range, IQR) for continuous variables and as frequency (%) for categorical variables. We used either two‐tailed *t* test/paired *t* test or the Wilcoxon rank‐sum test for comparison of two groups. ST‐2 change rate was calculated by subtracting pre‐ST‐2 from post‐ST‐2 and then dividing it by pre‐ST‐2. Multivariate linear regression analyses were used to assess the β and 95% confidence interval (CI) of cardiac dose parameters associated with ST‐2 change rate, with adjustment for major covariables including age, gender, smoking, history of coronary disease, diabetes mellitus, hypertension, chemotherapy, and surgery. The smooth curve fitting (penalized spline method) was used to characterize the shape of the associations between heart dose parameters and ST‐2 change rate. All analyses were performed using the statistical package R (http://www.R-project.org, The R Foundation) and Empower (R) (http://www.empowerstats.com; X&Y Solutions, Inc). A 2‐tailed *P* < .05 was considered statistically significant.

## 3 RESULTS

### 3.1 Population characteristics

Demographic data in the study are shown in Table 1. A total of 60 patients who received chest RT were enrolled, including patients with lung cancer (61.67%), esophageal cancer (30%), and thymoma (8.33%). The mean age was 61.5 years (range 30‐84). Among them, two had a history of coronary heart disease, five had diabetes mellitus, and 10 had hypertension. Of the 60 patients, 42 (70%) had received radical RT, 16 (26.7%) adjuvant RT, and 2 (3.3%) palliative RT.

**Table 1:** Characteristics of patients

Characteristic^a^	
Age	61.8 ± 9.6
Gender	
Male	52 (86.67%)
Female	8 (13.33%)
Smoking	
No	33 (55.00%)
Yes	27 (45.00%)
History of coronary disease	
No	58 (96.67%)
Yes	2 (3.33%)
Diabetes mellitus	
Without	55 (91.67%)
With	5 (8.33%)
Hypertension	
Without	50 (83.33%)
With	10 (16.67%)
Chemotherapy	
No	12 (20.00%)
Yes	48 (80.00%)
Surgery	
No	34 (56.67%)
Yes	26 (43.33%)
Type of pathology	
Lung cancer	37 (61.67%)
Esophagus cancer	18 (30.00%)
Thymoma	5 (8.33%)
Type of rt	
Radical	42 (70%)
Adjuvant	16 (26.7%)
Palliative	2 (3.3%)

Abbreviations: RT, radiotherapy.

a Data are presented as number (%) or mean ± standard deviation.

### 3.2 sST‐2 levels were elevated with the time of thoracic irradiation, while LVEF and BNP levels were not changed

**Figure 1:**
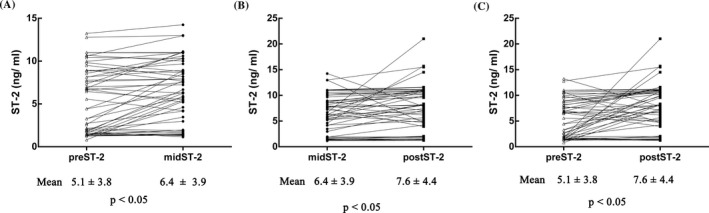
Serum ST‐2 level changes in different periods of RT. A, Distribution of sST‐2 levels between pre‐ST‐2 and mid‐ST‐2; B, Distribution of sST‐2 levels between mid‐ST‐2 and post‐ST‐2; C, Distribution of sST‐2 levels between pre‐ST‐2 and post‐ST‐2

We evaluated cardiac function changes using cardiac ultrasonography and serum cardiac biomarkers. Among the patients, the mean pre‐ST‐2, mid‐ST‐2, and post‐ST‐2 levels were 5.1 ± 3.8, 6.4 ± 3.9, and 7.6 ± 4.4, respectively. mid‐ST‐2 and post‐ST‐2 levels were significantly higher than pre‐ST‐2 levels (Figure 1).As shown in Figure 1, after RT and in comparison with pre‐ST‐2, mid‐ST‐2 level increased in 65.4% of patients; post‐ST‐2levels were increased in 71.2% patients. ST‐2 levels (75%) were elevated from mid‐ST‐2 to post‐ST‐2.

The median pre‐BNP, mid‐BNP, and post‐BNP levels were 22.56 (IQR 7.25‐63.5), 32.25(IQR 18.85‐52.97), and 32.41 (IQR 17.23‐55.97), respectively. However, there was no significant difference in BNP levels with the course of RT (Figure S1A). The mean pre‐LVEF and post‐LVEFwas 64.36 and 62.76, respectively (Figure S1B), with no significant difference (*P* > .05).

### 3.3 Association between heart dose parameters and levels of ST‐2

**Figure 2:**
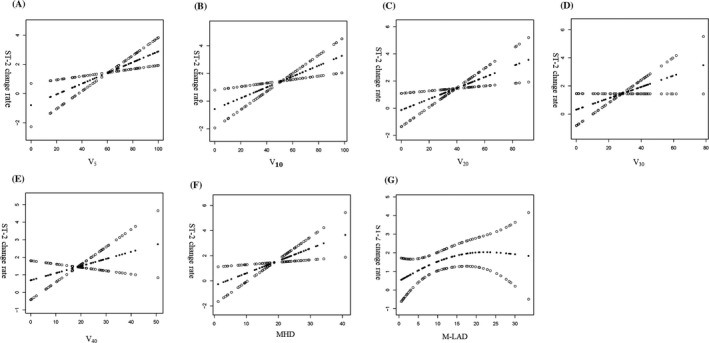
Association between heart dose parameters and ST‐2 change rate. A, V_5_and ST‐2 change rate; B, V_10_ and ST‐2 change rate; C, V_20_ and ST‐2 change rate; D, V_30_ and ST‐2 change rate; E, V_40_ and ST‐2 change rate; F, MHD and ST‐2 change rate; G, M‐LAD and ST‐2 change rate. The smooth curve fitting presented linear associations between cardiac dose parameters and ST‐2 change rate among patients with chest radiation. The solid black circle and empty circle represent the estimated values and their corresponding 95% CI

The mean V_5_, V_10_, V_20_, V_30_, V_40_, and MHD was 60.93 ± 27.79%, 51.43 ± 25.44%, 39.17 ± 21.75%, 28.07 ± 17.15%,18.66 ± 12.18%, and 18.60 ± 8.63 Gy, respectively. The median M‐LAD was 11.31 (IQR 3.33‐18.76) Gy. The association of cardiac dose parameters withST‐2 change rate, as assessed by multivariate linear regression analysis, is listed in Table 2. In the non‐adjusted model, V_5_, V_10_, V_20_, V_30_, and MHD were significantly and positively associated with ST‐2 change rate (*β* = .03, .04, .04, .04, and .09, respectively, all *P* < .05). After adjustment for age and gender, V_5_, V_10_, V_20_, V_30_, and MHD were independently and positively associated with ST‐2 change rate (*β* = .03, .03, .04, .04, and .09, respectively, all *P* < .05). In addition, after adjustment for all confounding factors including age, gender, smoking, history of coronary disease, diabetes mellitus, hypertension, chemotherapy, and surgery, V_5_, V_10_, V_20_, and MHD were still independently and positively associated with ST‐2 change rate(*β* = .04, .04, .04, and .1, respectively, all *P* < .05).Further analysis using smooth curve fitting(penalized spline method) confirmed that the association between heart dose parameters and ST‐2 change rate was linear (Figure 2).

**Table 2:** Relationship between heart dose parameters and ST change rate in different models

Variables	Non‐adjusted model		Adjusted model I		Adjusted model II	
	*β* (95%CI)	*P*‐value	*β* (95%CI)	*P*‐value	*β* (95%CI)	*P*‐value
V_5_	.03 (0.01, 0.06)	.0084	.03 (0.01, 0.05)	.0127	.04 (0.01, 0.06)	.0047
V_10_	.04 (0.01, 0.06)	.0081	.03 (0.01, 0.06)	.0106	.04 (0.01, 0.07)	.0055
V_20_	.04 (0.01, 0.07)	.0122	.04 (0.01, 0.07)	.0181	.04 (0.01, 0.07)	.0146
V_30_	.04 (0.00, 0.08)	.0441	.04 (−0.00, 0.08)	.0647	.04 (−0.00, 0.08)	.0572
V_40_	.03 (−0.02, 0.09)	.2285	.03 (−0.03, 0.09)	.2738	.04 (−0.02, 0.10)	.1893
MHD	.09 (0.01, 0.17)	.0257	.09 (0.01, 0.16)	.0341	.10 (0.02, 0.18)	.0187
M‐LAD	.05 (−0.02, 0.13)	.1913	.04 (−0.03, 0.12)	.2526	.05 (−0.03, 0.13)	.2419

Note

Non‐adjusted model adjusted for: None.

Adjusted model I adjusted for: age and gender.

Adjusted model II adjusted for: age, gender, smoking, history of coronary disease, diabetes mellitus, hypertension, chemotherapy, and surgery.

Abbreviations: CI, confidence interval; MHD, mean heart dose; M‐LAD, mean dose of left anterior descending artery; V_5_, volume of heart receiving ≥5 Gy, V_10_, V_20_ and so on.

## 4 DISCUSSION

We found that heart dose parameters in thoracic malignant tumor patients are associated with a change in ST‐2 change rate, when they received chest RT. Our results showed that compared with baseline, ST‐2 levels increased over time. However, compared with pre‐LVEF levels, post‐LVEF levels were not different, and the traditional cardiac biomarker BNP levels were also not changed. A positive association between heart dose parameters andST‐2 change rate was found.

Despite the rapid progress in cancer screening, diagnosis, and treatment, treatment‐related cardiovascular events such as radiation‐induced cardiac injury remain unavoidable.^5^ LVEF and blood markers (NT‐pro‐BNP/BNP and cTnI) are still classical methods in clinical practice for the risk assessment, diagnosis, and management of RIHD.^22^ In the small sample longitudinal study of cardiac biomarkers in patients receiving thoracic radiotherapy, Gomez et al^23^ showed that BNP increases during high‐dose irradiation of the heart in some patients. Recently, a long‐term retrospective study reported that median plasma BNP levels in 5‐year breast cancer survivors after radiation therapy remain within the normal range, but the delta‐BNP levels are positively related to the mean heart dose and mean left ventricular dose received.^24^However, the significance of BNP in the diagnosis and evaluation of radiation‐induced cardiac disease is not fully understood. Our results showed that BNP had not changed after RT, compared with BNP at the baseline, indicating BNP would not increase in short‐term post‐radiation therapy.

The left ventricle ejection fraction plays an important role in detecting cardiac function changes. Nousiainen et al^25^ demonstrated that early LVEF decline during doxorubicin therapy is associated with doxorubicin cardiotoxicity in lymphoma patients. However, Bianet al.^26^ found no acute changes in LVEF in breast cancer patients with concurrent trastuzumab and breast radiation. In this study, although the heart dose was higher than in the Bian et al study, the post‐LVEF levels were also not changed compared with baseline LVEF (pre‐LVEF) levels. Interestingly, we found that sST‐2 was increased during RT. Thus, sST‐2 might be useful in detecting acute or subclinical cardiotoxicity.

Accumulated results from clinical studies have shown that high cardiac radiation dose is directly associated with RIHDs.^24^, ^27^, ^28^, ^29^ Oncologists must also consider the rate of cancer control and the dose of cardiac radiation when formulating a RT regimen. In childhood cancer survivors, a large sample case control study revealed that heart failure often occurred in patients that received ≥30 Gy to the median volume of the heart.^30^ In stage III non‐small‐cell lung cancer, different cardiac events were associated with distinct heart volume doses; for example, ischemic events were correlated with left ventricle and whole heart dose.^27^ The mean V_5_, V_10_, V_20_, V_30_, V_40_, and MHD in our study was 60.93%,51.43%, 39.17%, 28.07%,18.66%, and 18.60 Gy. Our study demonstrated that V_5_, V_10_, V_20_, and MHD were independently and positively associated with ST change rate (*β* = .04, .04, .04, and .10, respectively, all *P* < .05).

We recognize that the study presents several limitations. First, this is a longitudinal study with a small sample size. Moreover, we collected serum samples only during and after radiotherapy, which mainly reflects acute radiation‐induced cardiac injury. Therefore, a long‐term follow‐up study with a large sample size is warranted. Fortunately, a registered clinical study 31 on early detection of RIHD is under way. Despite these limitations, this study analyzed the relationship between cardiac dose parameters and ST change rate for the first time in patients with chest radiation exposure and confirmed a linear relationship between them.

## 5 CONCLUSION

In conclusion, we showed that sST‐2 levels were elevated during radiotherapy over time in patients with thoracic malignant tumors when they received chest RT, and V_5_, V_10_, V_20_, and MHD were independently and positively associated with ST‐2 change rate.

## CONFLICT OF INTEREST

The authors declare that they have no conflict of interest.

## AUTHORS' CONTRIBUTIONS

Z‐M Z, in charge of design of the work, analysis, and wrote the article. P X, S Z, helped with acquisition and analysis of the data. X‐L J and J C helped to collect the serum samples. L H and A‐W L, the Corresponding author, was in charge of guidance of the design and analysis the whole research. All authors read and approved the final manuscript.

## ETHICS COMMITTEE

The study was consented by all the enrolled patients and the Ethics Committee of the second affiliated hospital of Nanchang University.

## Data Availability

The datasets used and analyzed during the study are available from the corresponding author on reasonable request, except private information of participants.
