# Supplementary figures and images for: Positive association between heart dosimetry parameters and a novel cardiac biomarker, solubleST‐2, in thoracic cancer chest radiation
Source: J Clin Lab Anal. 2020 Jan 10;34(4):e23150. doi: 10.1002/jcla.23150 (PMC7171349; doi:10.1002/jcla.23150)

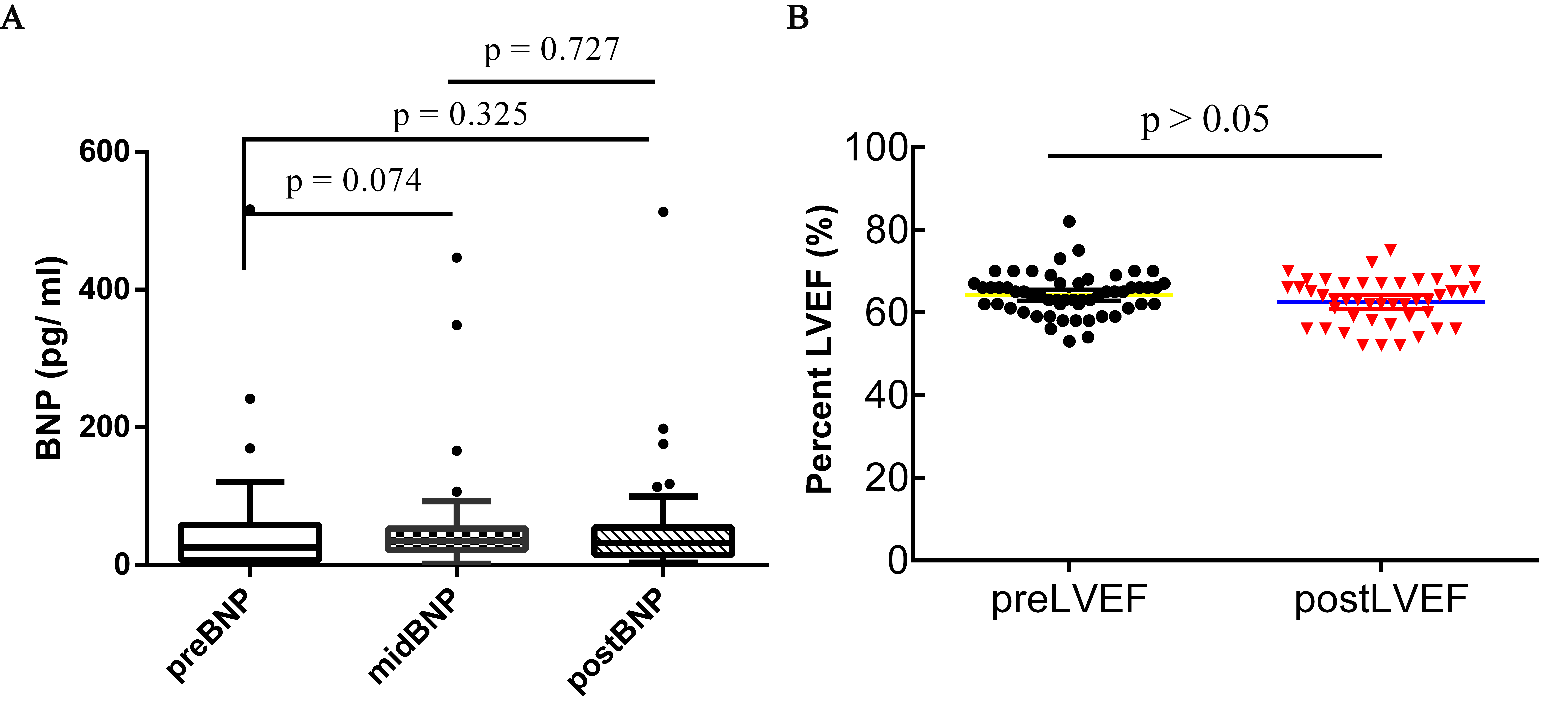

Supplement: Supplementary file 1 [file JCLA-34-e23150-s001.tif]
